# Differential expression of maize chitinases in the presence or absence of *Trichoderma harzianum *strain T22 and indications of a novel exo- endo-heterodimeric chitinase activity

**DOI:** 10.1186/1471-2229-10-136

**Published:** 2010-07-01

**Authors:** Michal Shoresh, Gary E Harman

**Affiliations:** 1 Department of Horticultural Sciences, Cornell University, Geneva, NY 14456 USA

## Abstract

**Background:**

The interaction of plants with endophytic symbiotic fungi in the genus *Trichoderma *alters the plant proteome and transcriptome and results in enhanced plant growth and resistance to diseases. In a previous study, we identified the numerous chitinolytic enzyme families and individual enzymes in maize which are implicated in plant disease resistance and other plant responses.

**Results:**

We examined the differential expression of the entire suite of chitinolytic enzymes in maize plants in the presence and absence of *T. harzianum*. Expression of these enzymes revealed a band of chitinolytic enzyme activity that had greater mass than any known chitinase. This study reports the characterization of this large protein. It was found to be a heretofore undiscovered heterodimer between an exo- and an endo-enzyme, and the endo portion differed between plants colonized with *T. harzianum *and those grown in its absence and between shoots and roots. The heterodimeric enzymes from shoots in the presence and absence of *T. harzianum *were purified and characterized. The dimeric enzyme from *Trichoderma*-inoculated plants had higher specific activity and greater ability to inhibit fungal growth than those from control plants. The activity of specific chitinolytic enzymes was higher in plants grown from *Trichoderma *treated seeds than in control plants.

**Conclusions:**

This is the first report of a dimer between endo- and exochitinase. The endochitinase component of the dimer changed post *Trichoderma *inoculation. The dimer originating from *Trichoderma *inoculated plants had a higher antifungal activity than the comparable enzyme from control plants.

## Background

*Trichoderma *spp. are opportunistic root colonizing fungal plant symbionts [1] that induce numerous changes in plant gene expression and physiology. Among the phenotypic changes are increased systemic resistance to plant diseases [1-8]; increased growth of plants and roots, including an increase in fertilizer use efficiency and uptake [9-12]; and a generalized increase in resistance to abiotic stresses [1,13].

We recently completed an analysis of the proteome of maize plants in the presence or absence of *T. harzianum *strain T22 [11,14]. Even though T22 was present only on roots, there were 141 proteins that we identified as up-regulated and 50 that were down-regulated in shoots [11], while in roots 20 up-regulated and 11 down-regulated proteins were found [14]. A large portion of the up-regulated proteins were involved in carbohydrate metabolism, while a number of others were involved in photosynthesis or resistance to stress. In addition, starch accumulation in maize plants whose roots were colonized with T22 was greater than in the control [11]. We suggested that *Trichoderma *induces both increased growth, which is mediated by an increase in photosynthetic and respiratory rates, and systemic induced resistance [14].

In an earlier study we determined that maize root colonization enhanced expression of chitinolytic enzyme activity [15]. This is frequently assumed to be part of induced systemic resistance [8]. However, in our proteomic studies, we did not identify up- or down-regulation of chitinolytic enzymes, probably primarily because we examined proteins with isoelectric points between 5.3 and 7.5, and the *pI*s of most chitinolytic enzymes are more basic or acidic than these levels. Prior to a recent study [16], only seven endochitinases and four partial sequences of exochitinases were identified. Using a combination of in silico and expression analyses, we identified putative 27 endochitinase genes (glycosyl hydrolases families 18 and 19) and four exochitinases (GH20; β-*N-*acetylhexosaminidases). The full sequences of these genes, domain analyses, and, in many cases, chromosomal locations were identified for these genes [16].

The chitinolytic enzyme families in plants and microbes are quite complex. They may cleave the chitin or other substrate molecules randomly or a single residue from the non-reducing end of the chitin molecule. These two types are commonly referred to as chitinases (endo-β-N-acetylglucosaminidase; EC 3.2.1.14) and exochitinases (β-N-acetylhexosaminidase; EC. 3.2.1.52). To avoid confusion, hereafter we will refer to the EC 3.2.1.14 enzymes as endochitinases and the 3.2.1.52 proteins as exochitinases, and any protein with enzymatic activity against chitin as a chitinolytic enzyme [17].

Given the great diversity of chitinolytic enzymes in plants, it is not very useful to simply quantify total chitinolytic enzyme activity as a quantitative measure of induced resistance or other factors. The total activity probably indicates little regarding the specific functionality of any individual gene product. In terms of disease resistance, as measured by antifungal activity, the differences between exo- and endo enzymes is quite large. In general, endochitinases have greater activity than exochitinases but mixtures of fungal endo and exo-acting enzymes usually are synergistic, and on a per unit protein basis, a combination of exo and endo enzymes usually are several-fold more active than any single enzyme [18,19].

In the course of our expression analyses of chitinolytic enzymes in maize with and without T22, we noted a chitinolytic enzyme band with a mass substantially larger than any expected gene product of the 31 different genes we identified in maize. This protein is a heterodimeric enzyme composed of an exo- and an endo-acting enzyme. Such heterodimers have not been heretofore described, and they may be of substantial importance since the heterodimeric enzyme would be expected to have substantially greater antifungal activity than any single protein. Moreover, the endo portion of the heterodimer differs in plants grown in the presence and in the absence of T22.

This paper describes the characterization of the differential expression of chitinolytic enzymes in maize with and without T22, the isolation of the heterodimeric enzyme from T22-treated and untreated plants and describes its very high antifungal activity.

## Results

### Chitin degrading enzymes of maize are different in shoots and roots and in the presence and absence of *T. harzianum*

**Figure 1:**
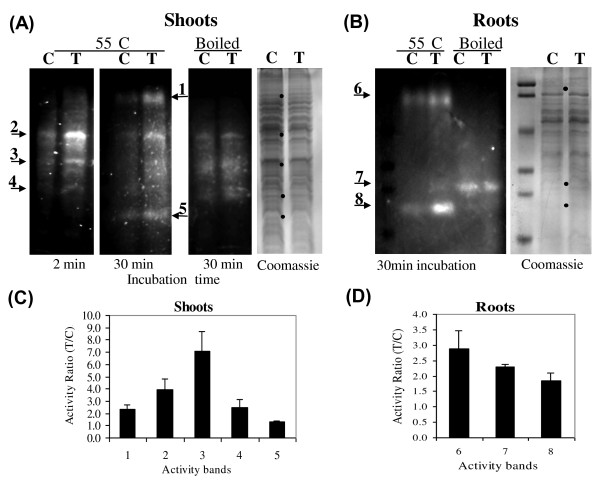
**Comparison of chitinase activity banding patterns of proteins extracts from control plants (C) and *Trichoderma*-inoculated (T) plants**. As described in the materials and methods, polyacrylamide gels with equal loading in each lane were removed from the glass plates following electrophoresis, subjected to various washes, and stained for chitinase activities using methylumbelliferyl substrates that were dissolved in 100 mM acetate buffer (pH 5.0) containing 1% low melting agarose. Chitinase activity bands from shoots (**A**) and roots (**B**) are shown. Activity bands started to appear as early as 2 min after beginning of incubation. Different intensities were observed for the activity bands in control and *Trichoderma*-inoculated plants, as shown in (**C**) -shoots and (**D**) -roots. The bands observed were numbered: 1-5 in shoots and 6-8 in roots; Bands intensities were measured and compared between the two treatments. Molecular size markers: 106, 93, 52, 32, 28, and 18 kDa.

Maize chitinolytic enzymes obtained from roots and shoots of plants grown from seeds treated or not treated with *T. harzianum *strain T22 were tested for activity in gels. Without boiling, five different activity bands were observed in shoots and two on roots. After boiling, the activity bands in shoots decreased to three, and the pattern changed in roots (Fig. 1). In both shoots and roots, activity of almost all bands was higher in the presence than in the absence of *T. harzianum *(activity of chitinolytic enzymes can readily be restored after boiling, as has long been known [20]). The only exception is band #5 which did not differ significantly between the two treatments (Fig. 1C and 1D). Activity band patterns observed in shoots were different from those observed in roots on the SDS-PAGE gels (Fig. 1A, B). Bands observed in gels exposed to the methylumbelliferyl substrates changed over time of incubation. Some activity bands of shoots appeared as early as 2 min after incubation while others appeared only later. After 30 min incubation the clarity of the earlier bands decreased due to diffusion of the fluorescent product (Fig. 1A). Bands #2, #3 and #4 (55 kDa, 43 kDa and 30 kDa, respectively) were observed as early as 2 min after incubation with the substrates. The rest of the bands appeared after 15 min of incubation. But bands #3 and #4 diffused by the end of 30 min incubation (Fig. 1A). Band #1 at the size of 95 kDa and band #5 at the size of 25 kDa appeared in the protein samples processed at 55°C but failed to appear when protein extracts of shoots were boiled before loading.

In roots, activity bands were observed to be 95 kDa and 23 kDa in both control and *Trichoderma*-treated plants in samples that were heated only to 55°C (Fig. 1B). However, no known chitinolytic enzymes have a molecular weight as large as 95 kDa. After boiling, only one activity band was observed at ca. 30 kDa. This suggested the possibility that the 95 kDa chitinolytic activity band is a complex of proteins.

Quantitative analysis of the fluorescence was performed using NIH-image software. Three independent repeats were performed and equal loading was used in each lane. In addition three major bands in each lane were quantified in the Coomassie stained images. These were used to verify equal loading and as a normalizing reference. In most cases the level of chitinolytic activity was 2- to 5-fold higher in plants grown from T22 treated seeds than from control seeds (Fig. 1C and 1D).

### Identification of chitinolytic enzymes

Thin slices of the activity bands were cut out of the gel and proteins were identified using LC/MS/MS. Mass-spectrometry results were screened against green-plant database into which the sequences of the maize chitinolytic enzymes identified in our previous study were incorporated [16]. Chitinolytic enzymes were identified in 10 bands at a confidence level higher than 95% (Table 1, for Enzyme nomenclature of these chitinolytic enzymes see Additional file 1). In band #6 of roots from T22 treated plants, Exo2 was identified at only 90% confidence. The parallel band from control plants also contained this enzyme (at a confidence level of 96%) suggesting that the identification of band #6 of root from *Trichoderma*-inoculated plants was correct.

**Table 1:** Identification of chitinases from the activity bands.

Plant treatment	Tissue	Protein treatment	Band	Identity	% Confidence
Control	Shoot	55°C	1	Exo2; chiIVA	96; 99
			
			2	No chitinase identified	
			
			3	No chitinase identified	
			
			4	chiIVB	99
			
			5	ChiI67336	99

*Trichoderma*	Shoot	55°C	1	Exo2; ChiIII9675	96; 96
			
			2	No chitinase identified	
			
			3	chiI11654	99
			
			4	No chitinase identified	
			
			5	ChiI67336	99

Control	Roots	55°C	6	Exo2; ChiIII9615	96; 98
			
			8	No chitinase identified	

*Trichoderma*	Roots	55°C	6	Exo2	90
			
			8	No chitinase identified	

Control	Roots	Boiling	7	chiIII9615	97

*Trichoderma*	Roots	Boiling	7	chiIII9615	99

Bands harboring chitinase activity were cut from gels and sent for identification by LC/MS/MS. The plant treatment and the tissues from which the proteins were extracted are listed. The proteins were either heated to 55°C or boiled before loading on gel. The numbers of the bands match the activity bands marked in Figure 1. The chitinases identified and the level of confidence is listed (when two proteins identified their % of confidence is listed respectively). The amino acid sequences, binding domains and other information for each of the proteins is described (Shoresh and Harman, 2008a).

Exo2 was the only protein identified in all tissues and treatments examined. ChiIVA and ChiIVB were identified only in shoots of control plants. ChiIII9675 and ChiI11654 were identified only in shoots from *Trichoderma *treated plants. ChiI67336 was identified in shoots of both control plants and those grown from seeds treated with *T. harzianum*. ChiIII9615 was identified in roots of both control plants and *Trichoderma *inoculated plants.

The molecular weights of the identified proteins fit their position in the gel supporting their identification, with the exception of the 95 kDa protein mentioned earlier. The LC/MS/MS data indicated that this band contained two enzymes; an endochitinase with a mass of a ca. 30 kDa and the other was Exo2 with a mass of 66 kDa (Table 1). This suggested that the protein in this band could be a heterodimer between Exo2 and an endochitinase. The endochitinase in this putative dimer differed according to the source of the tissue--the enzyme identified in shoots in the presence (ChiIII9675) and absence (ChiIVA) of T22 was different and it differed again between roots (ChiIII9615) and shoots of control plants (Table 1).

### Purification of the heterodimeric proteins from shoots

**Figure 2:**
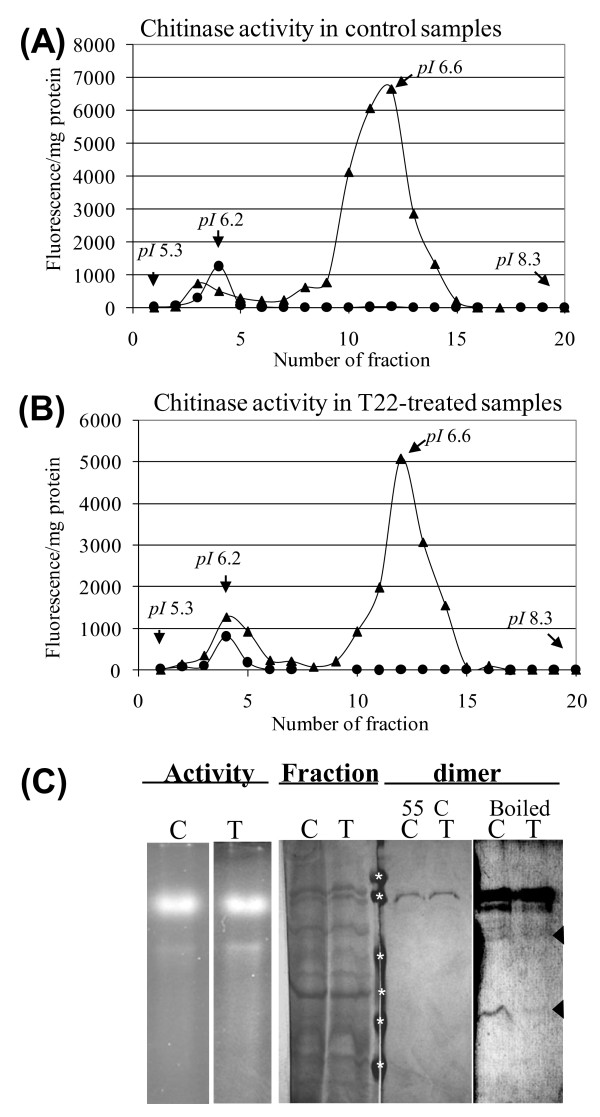
**Isolation of the protein dimers from shoots**. Chitinase activity profiles of the fractions collected following Rotofor purification from proteins obtained from control (**A**) or *Trichoderma*-treated (**B**) plants. The *pI *of several fractions is indicated. Triangles and circles are MUA and MUB tests, respectively. Gel analysis for the different stages of dimer purification is presents in (**C**). Activity staining for Rotofor fractions #4 from control and *Trichoderma*-treated plants (Native gels) is shown on the 1^st ^panel on the left; on the 2^nd ^panel is a coomassie staining of a sample of the same fractions run on SDS-PAGE. The active bands were sliced out and eluted from the native gels. A sample of these eluted proteins was analyzed on SDS-PAGE with protein-treatment of 55°C, silver stained and is shown on the 3^rd ^panel indicating that they are electrophoretically pure. On the 4^th ^panel (right side)- after boiling, the purified proteins dissociated into smaller proteins at the size of the components of the dimeric proteins as identified by LC/MS/MS (arrows). Molecular size markers (marked with asterisks): 106, 93, 52, 32, 28, and 18 kDa.

Proteins were isolated from shoots of control and *Trichoderma*-colonized plants and then were further separated into 20 fractions according to their *pI *using a Rotofor apparatus (Bio-Rad). Protein concentrations and chitinolytic activities were determined in each fraction and expressed as fluorescence per milligram protein using only MUA and MUB separately (Fig. 2A, B). In control samples only the third and fourth fractions contained activities on both MUA and MUB. In samples from plants colonized with *T. harzianum*, the fourth and fifth fractions contained enzymes that acted on both substrates. The combined activities in both types of tissues were in fractions at pH 6.2. The fourth fractions derived from control and *Trichoderma*-treated plants, which had the highest activity, were further analyzed.

The fractions indicated above were then separated on native PAGE gels. In-gel activity assays demonstrated a strong activity band in both control and *Trichoderma*-treated samples (Fig. 2C, Activity panel). SDS- PAGE gels of a sample from the fourth fractions revealed a number of protein bands (Fig. 2C, Fraction panel). The activity bands from the fourth fraction of the two treatments were cut out and extracted from the native gel. Portions of these extracted proteins were further run on SDS-PAGE gels and found to give a single band at the size of 95 kDa after silver staining (Fig. 2C, Dimer panel, 55°C lanes). The electrophoretically homogenous proteins thus obtained were then used for subsequent assays of enzymatic and antifungal activities, and are described as the purified heterodimeric proteins hereafter. The fold purification of the dimeric protein was tested using MUA and MUB. The MUB based specific activity increased 302- and 482-fold over the course of the purification for enzymes from the control and *Trichoderma*-treated plants, respectively (Table 2). The MUA based specific activity contained within the dimeric protein increased 2.8- and 5.1-fold in the control and *Trichoderma*-treated plants, respectively. The specific activities, based on the two substrates, of the purified heterodimer isolated from *Trichoderma*-treated plants were almost two-fold higher than the activity of the protein from control plants (Table 2).

**Table 2:** Specific activity of chitinase preparations

	MUB based Activity		MUA based Activity	
	
	Control	*Trichoderma *inoculated	Control	*Trichoderma *inoculated
Total extracted protein	0.033 ± 0.0069	0.035 ± 0.0037	0.40 ± 0.054	0.40 ± 0.04

Rotofor fraction 4	0.73 ± 0.0036	0.48 ± 0.0022	0.33 ± 0	0.73 ± 0.052

Purified dimeric protein	9.97 ± 0.198	16.88 ± 0.34	1.1 ± 0.0126	2.04 ± 0

Fold enrichment	302	482	2.8	5.1

Specific activity units are μM MU*min-1*μg-1. Measurements were performed as described in materials and methods using MUA and MUB separately. Known protein amounts from each preparation were taken for the assay.

In order to dissociate the dimers to their components the isolated dimers were boiled for 10 min in the presence of a reducing agent (50 mM TCEP·HCl). While boiling completely abolished activity, it did not completely dissociate the dimers into their components under these conditions. However, faint bands were observed at the sizes of 65 kDa and 30 kDa which are at the expected sizes of the exochitinase and the endochitinase identified by the LC/MS/MS from these 95 kDa proteins (Fig. 2C, Dimer panel, boiled). It is also worth noticing that the intensities of the dissociated bands were stronger for the dimer derived from control plants than the dimer derived from *Trichoderma*-treated plants. This may suggest that under these conditions dissociation of the control dimer is easier.

### Antifungal activity against *Penicillium digitatum*

**Figure 3:**
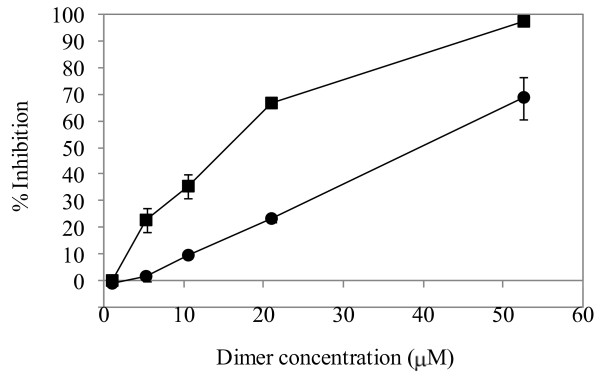
**Inhibition of Penicillium spore germination by the different dimers**. The percentage of spore germination inhibition is depicted versus the concentrations of the purified dimeric proteins. The error bars represent with standard deviations. Circles represent control dimer and squares represent dimer from *Trichoderma*-inoculated plants. The concentrations used were: 1.05, 5.26, 10.53, 21.05 and 52.63 nM for both dimers. Two independent experiments were performed. This quantitative analysis was used to calculate the IC_50 _of each dimer.

**Figure 4:**
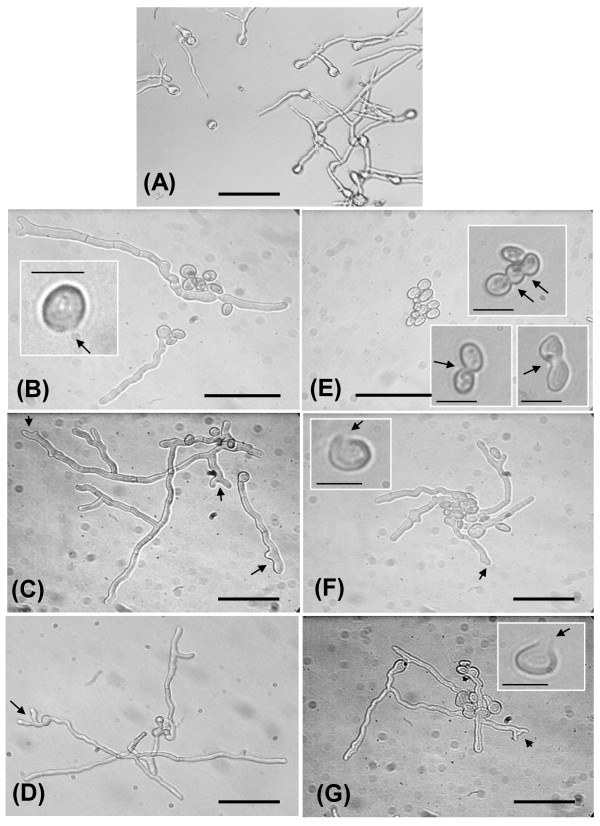
**Microscopic photographs showing the antagonistic effects of the different dimers on germination of *Penicillium digitatum *spores**. Different concentrations of the dimers were incubated with 1000 spores per well for 16-18 h and these were used to generate the photographs. (**A**) Control (extraction from empty gel); (**B**) Control dimmer- 52.63 nM, inset- enlargement of one spore with cell wall damage; (**C**) Control dimer - 21.05 nM, arrows indicate hyphal tip abnormalities; (**D**) Control dimer -10.53 nM, arrows indicate hyphal tip abnormalities; (**E**) Dimer from *Trichoderma *treatment- 52.63 nM, inset- enlargement of spores with cell wall damage and fusions; (**F**) Dimer from *Trichoderma *treatment- 21.05 nM, arrows indicate hyphal tip abnormalities inset-arrow point to damage of cell wall in one of the spores; (**G**) Dimer from the *Trichoderma *treatment- 10.53 nM, arrows indicate hyphal tip abnormalities, inset- enlargement of one spore with cell wall damage; Bars on pictures represent 35 μm and bars in insets represent 10 μm.

The antifungal activities of the purified heterodimeric proteins from shoots of control plants and of plants colonized by *T. harzianum *were compared. We tested the antifungal activity of the purified heterodimeric proteins in a model system utilizing *P. digitatum*. Both dimeric proteins inhibited spore germination (Fig. 3). However, the IC_50 _of the dimer from *Trichoderma *treated plants was 14.74 nM while the IC_50 _of the dimer from control plants was 38.95 nM. In addition to inhibition of spore germination, in the presence of 52.36 nM of dimer (from both control and *T. harzianum *treated plants) and at 21.05 nM and 10.53 nM of only the *T. harzianum *treated plants derived dimer, we observed degradation of the fungal cell walls in specific spots of some spores as well as fusion of spores, which suggests that they had been primarily converted to protoplasts (mostly seen in the *Trichoderma *treatment-derived dimeric protein) (Fig. 4B, E-G). At lower concentrations of both dimers, other abnormal structures were observed, such as swelling of the hyphal tips and branching of the very end of the hyphal tips. This relates to the weakening of the cell wall since the tip is more sensitive to cell wall degradation.

## Discussion

Chitinolytic enzymes in plants are numerous and highly diverse, including those in maize. The enzymes are arranged in various classes and into endo- and exo-acting enzymes based on domain analyses and sequence motifs. The classification of enzymes in Table 1 and in this paper is based on this sequence information; we recently have described the maize chitinolytic enzymes and published the sequences of the 27 endochitinases and four exochitinases from maize [16]. This study demonstrates that root colonization by *T. harzianum *not only results in changes in shoot and root chitinolytic enzyme activity levels as we have described earlier [15] but that qualitative changes also are induced. These data suggest that the chitinolytic enzymes in maize are under complex regulatory control and that *T. harzianum *induces changes in this regulation. Previous study has demonstrated that in our system *Trichoderma *is present only on roots [15]. Thus, observed changes in proteins in shoots are a consequence of systemic induction by *T. harzianum*, which has been noted in other studies as well [2,6,8,11,21,22]. Probably our assessment of the presence or absence of various chitinolytic enzymes is an underestimate, since we were able to identify only 10 of the activity bands.

There are so many chitinolytic enzymes, probably many with unknown physiological function, and therefore determination of total activity is not very useful. In many studies, only changes in total chitinolytic activity were measured [8,15]. In other cases, one, or at most a few, of the enzymes that may be produced in plant systems were studied [23-25]. A few studies have been more comprehensive; for example, a study on sugar cane pathogenesis examined four chitinases [26] and a study in rice examined distribution, structure, organ-specific expression and phylogenetic analysis of 12 chitinase III enzymes [27]. However, so far as we can ascertain, there are no fully comprehensive studies on the role of the total mixture of chitinolytic enzymes or genes in any plant process.

Two endochitinases, which were identified in the activity bands in this study, were previously shown to have antifungal properties [28]. Chitinolytic enzymes are no doubt important in pathogenesis [28,29,31-33]. Overexpression, or high level expression of heterologous chitinolytic enzymes, alone or in combination with other antifungal proteins frequently results in protection against pathogens [19,34-40]. The enhanced protection by the chitinases could be due to direct inhibition of fungal growth or due to induction of plant defense responses by the GlcNAc oligomers generated by their activity [41]. However, in other studies modulation of chitinase expression did not change the resistance of plants pathogens [42,43]. This suggests that chitinases may also have other roles (as demonstrated by [44,46-50]). Hence total activity of chitinases does not necessarily represent antifungal activity and a study of specific chitinases could greatly contribute to our understanding of their role.

Several *Trichoderma *strains induce the Induced Systemic Resistance (ISR) pathway [4,6] and others are suspected to induce the systemic acquired resistance (SAR) pathway [51]. These data suggest that different pathways of induced resistance may be activated by the presence of different strains. Expression profiles may be more complex when adding effects of pathogens in plants colonized or not colonized by *Trichoderma*. In cucumber, very different responses were identified in plants infected with the bacterial pathogen *Pseudomonas syringae *pv. *lachrymans *after root colonization by *T. asperellum *[8] than with the biocontrol agent alone. Thus, altered profiles of the complex mixture of chitinolytic enzymes are likely to be discovered by different combinations of *Trichoderma *strains and pathogens. It appears to be a very good system to examine the total potential of plants to express induced resistance and changes in chitinolytic enzymes can provide good markers for these differential responses.

In the course of the current research, we found an activity band with chitinolytic activity that was larger than the protein that would be expressed by any gene that we had identified. The molecular range of all identified maize chitinolytic enzymes is 55-66 kDa for exochitinases and 16-35 kDa for endochitinases. This indicated that none of these could be the sole constituent of this high molecular weight band. To our surprise LC/MS/MS identified an exochitinase, Exo2, together with different endochitinases, depending on the treatment and tissue type. The mass of this 95 kDa protein was equal to the sum of the exo- and endo-chitinases detected by LC/MS/MS and the activity of this band disappeared upon boiling. These data suggested that the 95 kDa protein was, in fact, a heterologous dimer between an exochitinase (Exo2) and an endochitinase (ChiIII9675 or ChiIVA, in the shoot). An exochitinase purified from mungbean was shown to exist in a hetero-dimeric form with an unknown protein [52]. Thus, we have identified novel heretofore unknown heterodimer comprised of an exochitinase and an endochitinase. Although the dimers were identified *in vitro *from plant extracts we hypothesize they may have an *in vivo *significance, for example, in plant defense.

We were intrigued by the appearance of the different endochitinase constituents in the dimer in the presence or absence of *T. harzianum *and their possible role in plant defense, especially since endochitinases and exochitinases were shown to possess synergistic antifungal activity in mixtures [19,34]. Since the specific chitinolytic activity of the dimeric protein in *Trichoderma*-treated plants is higher than from control plants, this suggested that the dimer from treated plants might possess higher antifungal activity. This was confirmed since the purified enzyme from shoots of *Trichoderma*-treated plants was about twice as effective in preventing germination of fungal conidia as was the corresponding protein from control plants. Similarly, lower concentrations of the *Trichoderma*-derived enzymes were required to cause obvious cell wall damage in germlings of *Penicillium digitatum*. There are no reports on ChiIII9675, which is one of the components of the *Trichoderma*-induced heterodimer, but the monomeric maize ChiIVA has antifungal activity [28]. There has been a great deal of interest in producing disease-resistant transgenic plants that express chitinases. For example, *Trichoderma *chitinases, when introduced transgenically into plants, induce resistance against a range of plant pathogenic fungi [19,34,35]. Although tested here only against one fungal model system further study of the dimers activity against other fungal pathogens is under way. Yet, the genes encoding these maize enzymes would appear to be good candidates for such uses since they are derived from a food plant, and therefore may be more acceptable than ones from microbial sources. The mechanisms by which *Trichoderma *spp. induce plants to be more resistant to disease is just now being understood. It could be that the change in chitinase activity profile and the formation of dimers with higher activity are part of this mechanism. It is likely, but unknown, whether these dimers exist in other plants. The *in vivo *role of these dimeric chitinase proteins in inducing resistance in plants to fungal attack deserves further attention.

## Conclusions

Using chitinase activity assays and gel-based proteomic approach we characterized the differential expression of the entire suite of chitinolytic enzymes in shoots and roots of maize plants in the presence and absence of *T. harzianum*. We further isolated heretofore undiscovered heterodimeric proteins which include an endo- and exochitinase constituents. Change of the endochitinase component of the dimer resulted in change of the enzymatic activity.

## Materials and methods

### Plant and fungal material

Seeds of maize (*Zea mays *L.) inbred Mo17 were treated with *Trichoderma harzianum *Rifai strain 22 (T22) in a cellulose-dextran formulation (1-2 × 10^9 ^cfu/g) [53] or were treated with water. Previous work with application of the cellulose-dextran powder without T22 gave no observable difference than water application (data not shown). The cellulose-*Trichoderma *powder was suspended in water (38.5 mg/5 ml) and 100 μl were applied to 5g of seeds. Seeds were planted in sandy loam field soil in boxes (10.5 × 10.5 × 6 cm) with five seeds per box. Seed treatments with T22 result in colonized roots, but the organism does not grow on or in shoots [15]. Boxes were incubated in a growth chamber with diurnal fluorescent lighting with 16 h/8 h (light/dark cycle), at 22 ± 4°C, and watered as needed. Seven-day-old seedlings were harvested: the shoots were first measured for heights and then excised 1 cm above soil level, frozen immediately in liquid nitrogen and stored at -70°C until use.

### Protein extraction

Shoot tissue samples were ground with liquid nitrogen followed by further grinding in 9 ml of ice cold 0.1 M HEPES and 2% dithiothreitol (DTT) per 3 g tissue powder using an Ultra-Turrax homogenizer (Janke & Kunkel). Two repeats with a total of about 10 g shoot tissue each were processed for each treatment. For ca. 10 g of control plants, 55 plants were used. For *Trichoderma*-treated plants, 40 plants were used to get ca. 10 g of tissue. The homogenate was then centrifuged for 20 min at 15000 pm at 4°C. Proteins were precipitated from the supernatant by adding 8 volumes of ice-cold acetone and incubating 16 h at -20°C. After another centrifugation the precipitated proteins were washed twice with 2 ml of ice-cold acetone followed by drying under a flow of N_2_. Powder was than dissolved in sample solubilization buffer (100 mM sodium acetate pH5.5, 10 mM EDTA, 0.1 mM DTT). A small aliquot was diluted 50-fold with water and the protein content was determined using Coomassie Plus Protein Assay (Pierce) according to manufacturer's instructions.

### PAGE and in-gel chitinase activity assay

SDS-polyacrylamide gel electrophoresis (SDS-PAGE) was employed to assess the relative amounts and banding patterns of the chitinases in protein samples. Protein samples were mixed with 25% by volume of loading dye (15% sucrose; 2.5% SDS; 125 mM Tris-HCl, pH 6.7; 0.01% Bromo-phenol Blue), loaded on a 4% acrylamide stacking gel and separated in a 12% acrylamide gel using a Mighty Small II electrophoresis system (Amersham). Gels were then used for in-gel chitinase activity or stained with Coomassie or silver stain using standard procedures.

Basic-native PAGE (native-PAGE) was used to isolate the protein from the piece of gel harboring the chitinase activity assay. Protein samples were mixed with 1/5 of loading dye (100 mM of Tris-HCl, pH 6.8; 50% glycerol; 0.01% Bromo-phenol Blue) and were loaded on a 4% acrylamide stacking gel with 50 mM of Tris (pH 6.8) and separated using a 10% acrylamide gel with 360 mM of Tris-HCl (pH 8.9). The running buffer contained 50 mM Tris-Base and 380 mM glycine, pH 8.9. Proteins were separated for about 5 h at a constant 30 mA/gel with cooling.

Before the in-gel chitinase activity assay, native gels were washed with acetate buffer (100 mM, pH 5.0) for 20 min. SDS-gels were washed three times in renaturation buffer (40 mM Tris-HCl, pH 9.0; 2 mM EDTA; 1% casein) for 30 min per wash and then washed for 20 min in 100 mM sodium acetate buffer (pH 5.0) with 1% (v/v) purified Triton-X100. This was followed by two washes with 100 mM sodium acetate buffer (pH 5.0) for 15 min each. Similar procedures have long been used to renaturate chitinolytic enzymes following treatments appropriate for SDS-PAGE gels (Trudel and Asselin, 1989).

Gels were then stained for chitinase activity by overlaying with 1% low melting (≤35°C gelling temperature) agarose that contained methylumbelliferyl substrate in 100 mM acetate buffer (pH 5.0). Substrates used were 4-methylumbelliferyl-*N*-acetyl-β-D-glucosaminide (MUA; 20 ng/ml), 4-methylumbelliferyl-*N*-acetyl-β-D-*N, N', N"*-triacetylchitotrioside (MUB; 16.6 ng/ml), 4-methylumbelliferyl-*N*-acetyl-β-D-*N, N'*-diacetylchitobioside (MUC; 10 ng/ml) and 4-methylumbelliferyl *N*-acetyl-β-D-*N, N', N"N'''*-tetraacetylchitotetraoside (MUD; 10 ng/ml) (Sigma, St. Louis, MO). Agarose was melted in a microwave oven and kept in a water bath at 37°C and substrates were added prior to application. Gels were kept at room temperature and the activity bands were observed after 2, 15 and 30 min under UV light. Following photo imaging of chitinase activity banding patterns, the agarose layer was gently removed from gel and gels were rinsed in deionized water and stained for protein profiles by coomassie or silver staining using standard procedures.

For dissociation of dimers, tris(2-carboxyethyl) phosphine (TCEP·HCl) was added to a final concentration of 50 mM with the sample buffer and protein samples were boiled for 10 min before loading on the gel.

### Rotofor separation

Proteins were separated according to their *pI *using a Rotofor Cell (Bio-Rad) according to manufacturer's instructions. Before separation proteins were dissolved in 2% ampholyte (Bio-Lyte 5/8, Bio-Rad) and 1% purified Triton X-100. Twenty fractions were collected and the pH of each fraction was recorded. Proteins were precipitated with addition of 8 volumes of cold acetone and incubated 16 h at -20°C. After 20 min of centrifugation at 15000 rpm at 4°C, pellets were washed twice with cold acetone and dissolved in 250 μl of 100 mM sodium acetate (pH 5.0). Three μl were diluted 1:10 in water and the dilutions were used to measure protein concentration and chitinase activity (in 96-well plates).

### Chitinolytic enzyme activity assay in plates

Chitinolytic activities were quantified in 96-well plates. In each well 10 μl of BSA, 30 μl of tested sample (at the desired dilution) and 30 μl of substrate (0.2 mM of either MUA or MUB) were added, keeping the plate on ice. The plate was then covered, sealed in a plastic bag and incubated for 30 min at 37°C. The reaction was stopped with 30 μl of 1 M sodium carbonate and the fluorescence was determined at 360/460 (excitation/emission) with a CytoFluor II fluorescence multiwell plate reader. Fluorescence of known concentrations of 4-methylumbeliferone (Sigma) was used to plot a standard curve to determine the activity of the chitinase in samples tested. The activity was defined as micromolar methylumbeliferone released per min per microgram of protein.

### Extraction of proteins from acrylamide gels

Activity bands were cut out of the native gels (care was taken to obtain pieces that were as narrow as possible). Gel bands were mashed through a nylon mesh (SpectraMesh poly; Spectrum Medical Industries, Los Angeles, CA). After addition of 100 μl water, the gel was left overnight at 4°C with occasional mixing. Mashed gels were loaded on glass fiber columns (3 mm height) and centrifuged at maximum for 5 min. The eluted protein was transferred to a new tube and 50 μl of water were added to the column. After incubation of 1 h at room temperature, centrifugation was repeated.

### Mass-spectrometry analysis and protein identification

Proteins were identified by peptide sequencing using nanospray ion-trap tandem mass spectrometry (nESI-IT MS/MS). The nESI-IT MS/MS experiments were performed on an LC Packings (Dionex)/4000 Q Trap (Applied Biosystems) in positive ion mode. Protein identification was carried out using the PMF - GPS Explorer, ESI - Analyst (Applied Biosystems) software. Non-redundant NCBI (National Center for Biotechnology Information, W) and SwissProt (European Bioinformatics Institute, Heidelberg, Germany) databases as well as our local chitinase sequences database were used for the search. Searches were performed in the full range of Mr and pI. Positive identification was considered only for C.I.% of ≥95%.

### Antifungal activity

Antifungal assays were conducted in 96-well plates using a standard assay [54]. Each well contained a spore suspension of *Penicillium digitatum *(1000 spores per well) and purified dimer at different concentrations in a final volume of 30 μl in 1/3 strength PDB (potato dextrose broth, Difco). Extraction from an empty gel was performed and this solution was added in place of enzyme solutions for the control. The microplates were incubated for 16-18 h at 25°C. Observations following incubation were taken directly from the microplate wells under a Nikon Diaphot brightfield microscope (Nikon Inc., Melville, NY, U.S.A.). The percentage of conidia germinated was determined based on a screen focused to the center of the well. Microscope slides were also prepared for observation at higher levels of magnification. Abnormal mycelial growth and morphological anomalies such as branching, bursting, appearance of necrotic zones, and lysis of the hyphal tips were recorded and photographed. The assay was repeated independently on two separate days.

## Authors' contributions

MS conducted all experimental methods and data analysis, MS and GEH designed research and wrote the manuscript. All authors read and approved the final manuscript.

## Supplementary Material

Additional file 1 **Supplemental Table**. Enzyme nomenclature of the chitinolytic enzymes described in this study. Click here for file
