# Supplementary material for: Differential expression of maize chitinases in the presence or absence of Trichoderma harzianum strain T22 and indications of a novel exo- endo-heterodimeric chitinase activity
Source: BMC Plant Biol. 2010 Jul 1;10:136. doi: 10.1186/1471-2229-10-136 (PMC3017806; doi:10.1186/1471-2229-10-136)
Supplement: Additional file 1 — Supplemental Table. Enzyme nomenclature of the chitinolytic enzymes described in this study. [file 1471-2229-10-136-S1.DOC]

**Additional file 1.**

**Supplemental Table:** Enzyme nomenclature of the chitinolytic enzymes described in this study.

| **Chitinolytic enzyme** | **EC number** | **CAZY nomenclature** |
| --- | --- | --- |
| chiIII9675 | 3.2.1.14 | Glycoside Hydrolase Family 18 |
| chiIII9615 | 3.2.1.14 | Glycoside Hydrolase Family 18 |
| chiI67336 | 3.2.1.14 | Glycoside Hydrolase Family 19 |
| chiI11654 | 3.2.1.14 | Glycoside Hydrolase Family 19 |
| chiIVA | 3.2.1.14 | Glycoside Hydrolase Family 19 |
| chiIVB | 3.2.1.14 | Glycoside Hydrolase Family 19 |
| Exo2 | 3.2.1.52 | Glycoside Hydrolase Family 20 |
